# Supplementary material for: Causal Relationship between Aging and Anorexia Nervosa: A White-Matter-Microstructure-Mediated Mendelian Randomization Analysis
Source: Biomedicines. 2024 Aug 16;12(8):1874. doi: 10.3390/biomedicines12081874 (PMC11351342; doi:10.3390/biomedicines12081874)
Supplement: Supplementary file 1 [file biomedicines-12-01874-s001.zip › Supplementary Figure/Figure S7/Single SNP plot of MR analysis for ubm-b-1957 to AN.pdf]

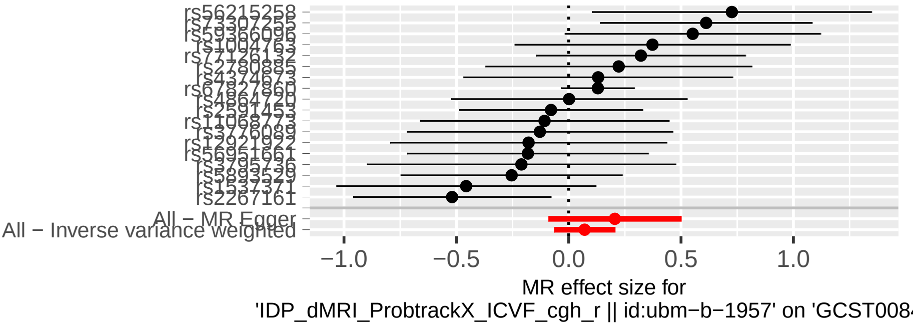
rs56215258
rs73307255
rs59366096
rs1004763
rs77126132
rs2780885
rs4374673
rs67827860
rs4864720
rs2591453
rs11068773
rs3776089
rs12921922
rs56951661
rs3795736
rs5893529
rs1537371
rs2267161
All − MR Egger
All − Inverse variance weighted
−1.0
−0.5
0.0
0.5
1.0
MR effect size for
'IDP_dMRI_ProbtrackX_ICVF_cgh_r || id:ubm−b−1957' on 'GCST008
